# Comparison of mechanical properties in interference screw fixation technique and organic anterior cruciate ligament reconstruction method: a biomechanical study

**DOI:** 10.1186/s12891-021-04788-3

**Published:** 2021-12-20

**Authors:** Amirhossein Borjali, Amir Nourani, Hadi Moeinnia, Mahdi Mohseni, Hossein Korani, Narges Ghias, Mahmoud Chizari

**Affiliations:** 1 Department of Mechanical Engineering, Sharif University of Technology, Tehran, Iran; 2 School of Mechanical Engineering, College of Engineering, University of Tehran, Tehran, Iran; 3 Department of Engineering and Technology, University of Hertfordshire, College Lane Campus, Hatfield, AL10 9AB UK

**Keywords:** ACL reconstruction, BASHTI technique, Core bone, Implant-less, Interference screw, Tensile test

## Abstract

**Background:**

Bone and Site Hold Tendon Inside (BASHTI) technique is an organic implant-less technique for anterior cruciate ligament (ACL) reconstruction with some clinical advantages, such as speeding up the healing process, over implantable techniques. The study aims to compare the mechanical properties of BASHTI technique with the conventional interference screw technique.

**Methods:**

To investigate the mechanical properties, 20 in-vitro experimental tests were conducted. Synthetic dummy bone, along with fresh digital bovine tendons, as a graft, were used for experiments. Three loading steps were applied to all specimens, including a preconditioning, a main cyclic, and a pull-out loading.

**Results:**

The mechanical characters of an interference screw technique using an 8 mm tendon diameter, including fixation strength, average cyclic stiffness (ACS), and average pull-out stiffness (APS) were found to be 439 ± 132 N, 10.3 ± 5.3 kN/mm, and 109 ± 40 N/mm, respectively. In the case of an interference screw using a 9 mm tendon, the fixation strength, ACS, and APS were obtained 549 ± 87 N, 10.3 ± 4.7 kN/mm, and 91 ± 13 N/mm, respectively. In parallel, the fixation strength, APS, and ACS of BASHTI technique using an 8 mm tendon were 360 ± 123 N, 3.3 ± 0.6 kN/mm, and 79 ± 27 N/mm, respectively, while, for 9 mm tendon 278 ± 103 N, 2.4 ± 1.2 kN/mm, and 111 ± 40 N/mm, were reported for fixation strength, APS, and ACS respectively when BASHTI technique was used.

**Conclusion:**

About 50% of interference screw samples showed superior mechanical properties compared to BASHTI technique, but in another half of the samples, the differences were not significant (N.S.). However, due to organic advantages of BASHTI technique and lower cost, it could be used as a substitute for interference screw technique, especially where fast recovery is expected.

## Abstract (article highlights)

- Developing an organic implant-less technique which has numerous clinical advantages
- Comparing this novel technique with interference screw technique
- Implementing synthetic Sawbones blocks to have a controlled study
- Selecting the best fixation condition for this novel technique

## Background

Knee is the joint which encounters significant injuries in the human body. Recent research shows that about 40% of all ligamentous knee injuries are related to anterior cruciate ligament (ACL) injuries, while about 70% of ACL injuries occur during sports activities [1]. As a matter of fact, an ACL injury is a non-contact injury that is in association with sports activities [2, 3].

When the ACL is completely torn, due to a lack of self-healing process in the ligaments [2, 3], the reconstruction surgery should be used [4]. The Swedish National ACL Register reported for about half the ACL injuries reconstruction is needed. Also, in the USA, probably 100,000–200,000 ACL reconstructions are performed each year [5, 6].

Among various techniques in ACL reconstruction, using interference screw is the gold standard technique [7, 8]{Sarzaeem, 2014 #392}. In this technique, different types of grafts could be used as a substitute for a torn ACL. Using bone-patellar tendon-bone (BPTP) graft and hamstring graft for ACL reconstruction are more conventional. Though, the use of interference screw implants in ACL reconstructions may cause some issues. One of the main problems of using interference screw methods is the high cost of these implants. Moreover, the use of interference screw may come with some side effects such as tunnel widening [9, 10], graft rotation [11], inflammatory reactions and abscesses production [12, 13], abrasion [14], interference in imaging after surgery [15], graft damage especially when using hamstrings tendon [16], and the risk of corrective surgery.

Bone and Site Hold Tendon Inside (BASHTI) technique is a recent organic implant-less technique that has minimum side effects compared to both the interference screw technique and the press-fit method. BASHTI technique was proposed at Sharif University of Technology in 2015 [17]. This technique is used to fix a hamstring graft into a bony tunnel. The fixation process in BASHTI technique is like the interference screw technique, but instead of the interference screw, the patient’s own bone is used to fix the tendon graft into the tunnel. In this regard, a cannulated drill bit is used to harvest a core bone by tunneling either the tibia or femur bone. Afterwards, the graft passes through the tunnel, and the core bone pushes back into the tunnel using hammer strikes. Therefore, there would not be the aforementioned problems when external implants are used. More importantly, in BASHTI technique, the bone to bone interface would speed up the healing process [7, 18].

Since the reconstruction technique is highly dependent on the bone quality of the patients, the effect of the bone density on the fixation strength was investigated in Dehestani’s study [19]. To do so, a range of polyurethane foam blocks from Sawbones (Pacific Research Laboratories, Malmo, Sweden) was used. Each Sawbones block corresponded to a specific group age of people. The results showed BASHTI technique is more appropriate for middle-aged and especially young patients [19]. In another research, the effect of sheathed core bone on the fixation strength of BASHTI technique was examined [20]. This technique made the BASHTI fixation process become more feasible and, also, increased the fixation strength of this technique [20]. Furthermore, since the geometrical variation on the mechanical properties has a significant impact, the geometrical parameters on the fixation strength of BASHTI technique were studied [21–24]. The results showed that the mode of the tendon failure was highly influenced by the tendon diameter and core bone diameter. Lastly, in a study, the insertion frequency in BASHTI technique was evaluated. It was suggested that insertion frequency of fewer than 300 beats per minute resulted in a safer core bone insertion and higher fixation strength [25].

Bashti et al. [17] compared the fixation strength of BASHTI technique with that of the interference screw method using in-vitro experiments. The tests were carried out using bovine bones and Achille tendons harvested from bovine feet. The results showed there was no significant difference between BASHTI and interference screw techniques in terms of fixation strength [17]. However, the bone samples used for this study were bovine bones, and they had different material properties in comparison with human bone. Since the BASHTI technique is highly dependent on bone density, the tested results on bovine bones may differ from those of human bones. Also, the samples were not tested in controlled groups; e.g. the pull-out rate, which may have a significant impact on the results, was not determined. Besides, the reports in this study did not include some significant mechanical properties of these kinds of tests, e.g., average cyclic stiffness and average pull-out stiffness. Lastly, the geometrical variations, which plays a crucial role in the mechanical properties of BASHTI technique, were not considered.

The current study aimed to compare the gold standard interference screw ACL reconstruction with BASHTI technique in order to have a clear understanding of the functionality of BASHTI technique in a controlled study. This study investigated all necessary mechanical properties affecting the fixation strength of the structure. The comparison was performed in controlled groups with specific graft sizes that resulted in the best fixation strength in BASHTI technique [21, 24]. It is hypothesized that the comparison would reveal clinical possibility of the BASHTI technique compared to the interference screws method.

## Methods

### Preparation of graft samples

**Fig. 1:**
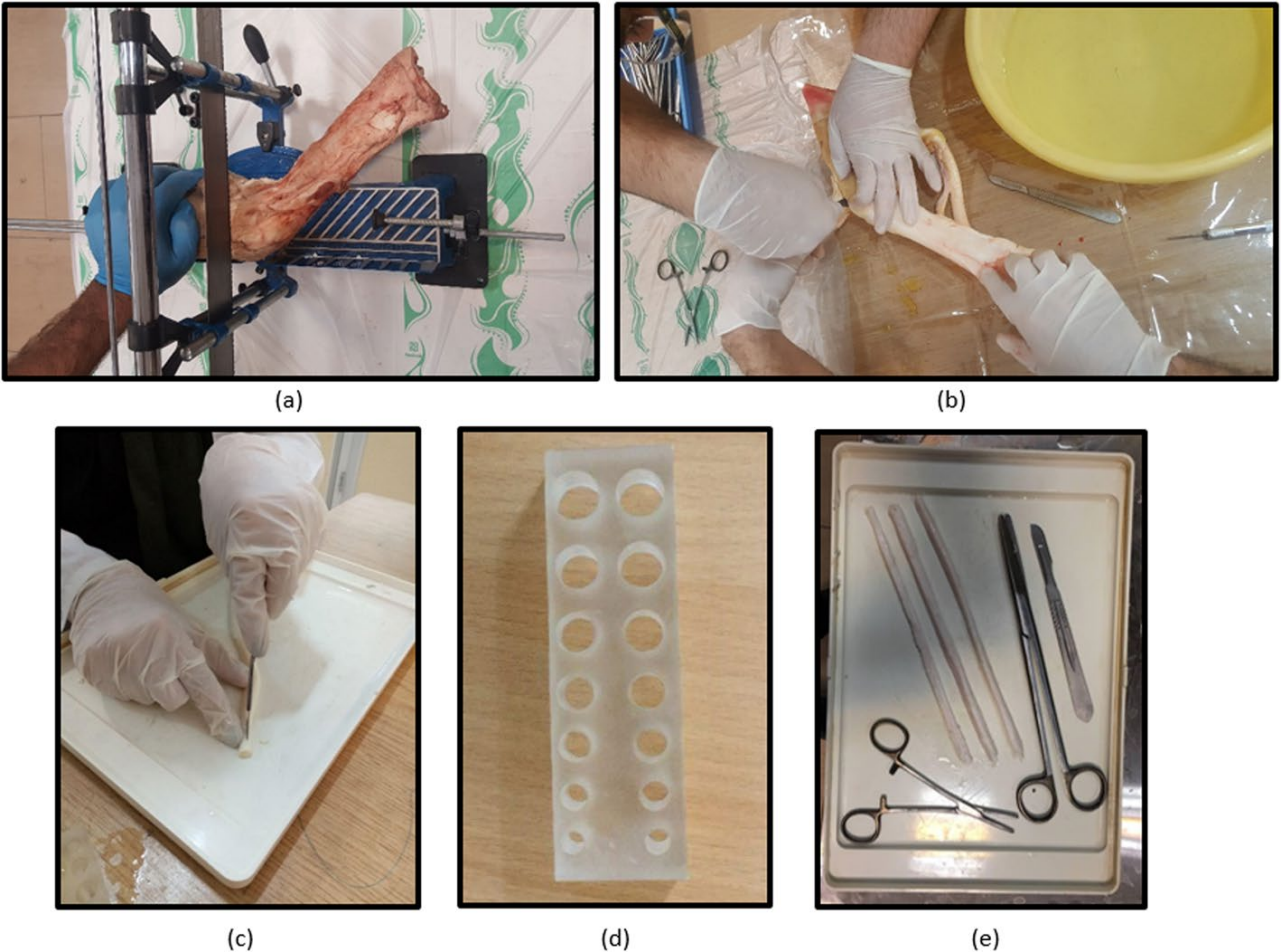
Harvesting a graft from bovine extensor/flexor tendons; **a** Cutting the bone feet into the desired part. **b** and **c** Trimming the tendon to a desired size. **d** A gauge template to evaluate the size. **e** The final grafts that are ready to test with the tools used in preparation

Bovine digital tendons were harvested from fresh bovine feet of the same race and age. Studies showed that the mechanical properties of bovine digital extensor tendons had no considerable differences with human hamstring tendons considering the range of loading applied on an ACL reconstruction [26]. Consequently, bovine digital tendons were used in laboratory conditions as a substitute for hamstring tendons. The grafts were harvested immediately after the animals were sacrificed. Both digital extensor and flexor tendons were harvested and trimmed to 8 and 9 mm diameters in a double-stranded fashion. In order to check the diameters carefully, the looped tendons were passed through a gauge template with appropriate hole diameters (Fig. 1d). The process of harvesting graft is shown in Fig. 1. It is noteworthy that the bovine tendons harvesting procedure, restrictedly, was performed under a local ethical and clinical approval, and bovine feet were stored according to the local food health and safety protocols.

Tendon graft samples were held in a freezer at − 20 °C as storing the tendon materials at this temperature for less than 48 h, has been proven to have no significant effect on the mechanical properties of the samples [27, 28]. At the time of testing, samples were exposed at room temperature and kept moist using a saline spray to retain their mechanical properties [27–29].

### Artificial bone blocks

Polyurethane foam blocks from Sawbones Company (Pacific Research Laboratories, Malmo, Sweden) were used to have a controlled study. The density of these Sawbones blocks was selected considering the material properties of the human bone. Sawbones blocks have similar material properties (e.g., density, Young’s modulus, and Poisson’s ratio) compared to actual bone samples [30]. Dehestani et al. showed BASHTI technique is more suitable for young patients. They found that Sawbones with a density of 320 kg/m^3^ well represented the proximal tibia bone quality of young people [19]. As a result, Sawbones blocks with the size 130 mm × 180 mm × 40 mm, and a density of 320 kg/m^3^ were used in the experiments.

### Cutting Core bone plugs

**Fig. 2:**
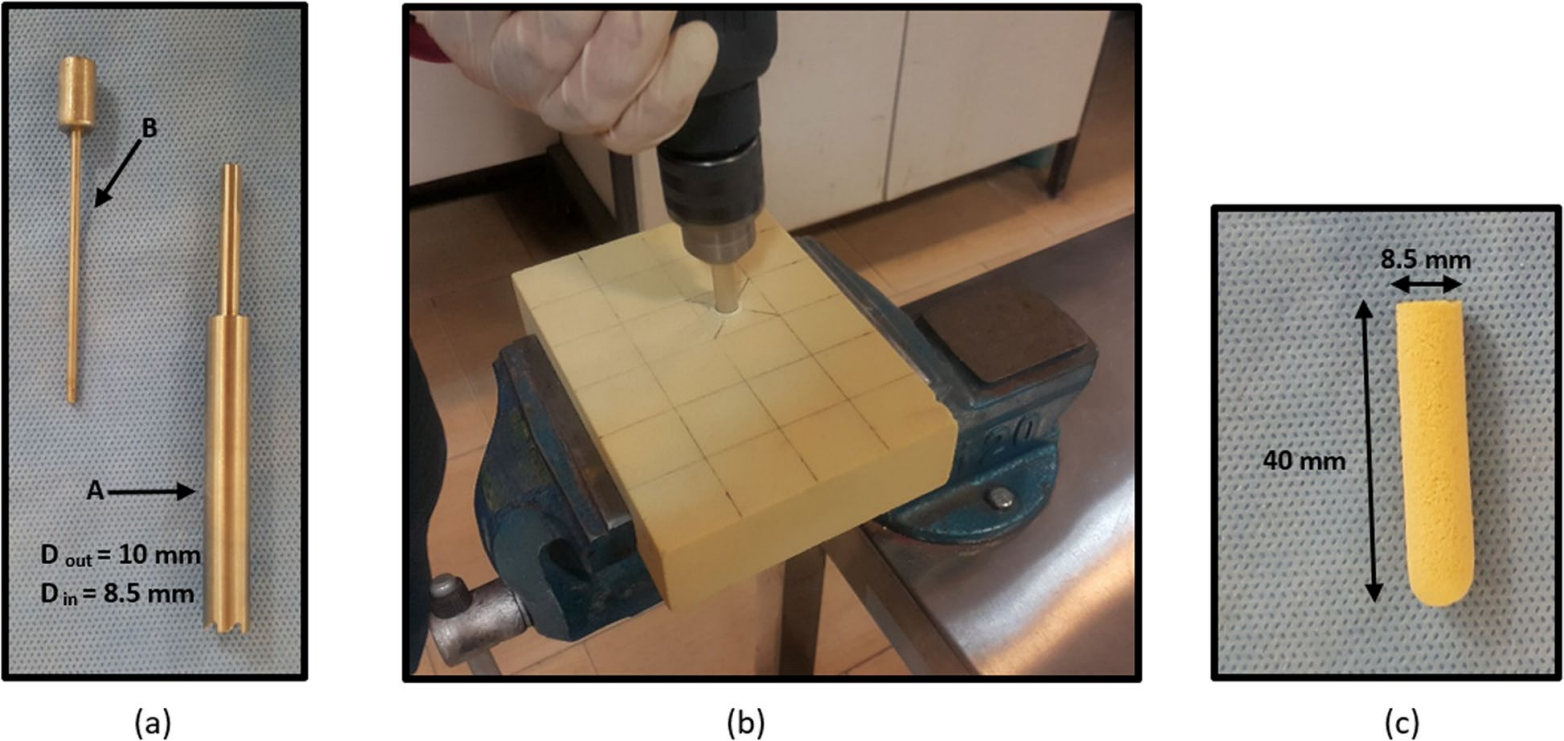
**a** Custom-made cannulated drill bit. This drill bit consists of two parts. The main body is used to create a tunnel (A), and the pusher is used to extract the core bone out of the cannulated drill bit (B). **b** Drilling process of the Sawbones block. **c** A typical core bone extracted from the tunnel with 8.5 mm diameter

In the next step, a cylindrical plug was cut and extracted from the block to create a core bone and a tunnel. To do so, a custom-made cannulated drill bit was designed and fabricated at Sharif Biomechanics Lab, Sharif University of Technology (Fig. 2a). Since the drill bit had a specific wall thickness with inner and outer diameters, when the tunnel was created on the block using this cannulated drill bit, a core bone with the size of its inner diameter would be extracted, while, the tunnel size is the same as the outer diameter of the cannulated drill bit. Then, this core bone plug was used to fix the graft into the tunnel. The inner and outer diameters of the cannulated drill bit were 8.5 and 10 mm, respectively. Therefore, a core bone with an 8.5 mm diameter was extracted from the tunnel of 10 mm diameter. The size of the tunnel and core bone was based on the previous study, which proved that this geometry yielded the best outcome and mechanical properties in the BASHTI technique [21]. Also, the length of the core bone plug was 40 mm, which was equal to the thickness of the Sawbones block. The drilling process with the cannulated drill bit is shown in Fig. 2.

### BASHTI insertion process

**Fig. 3:**
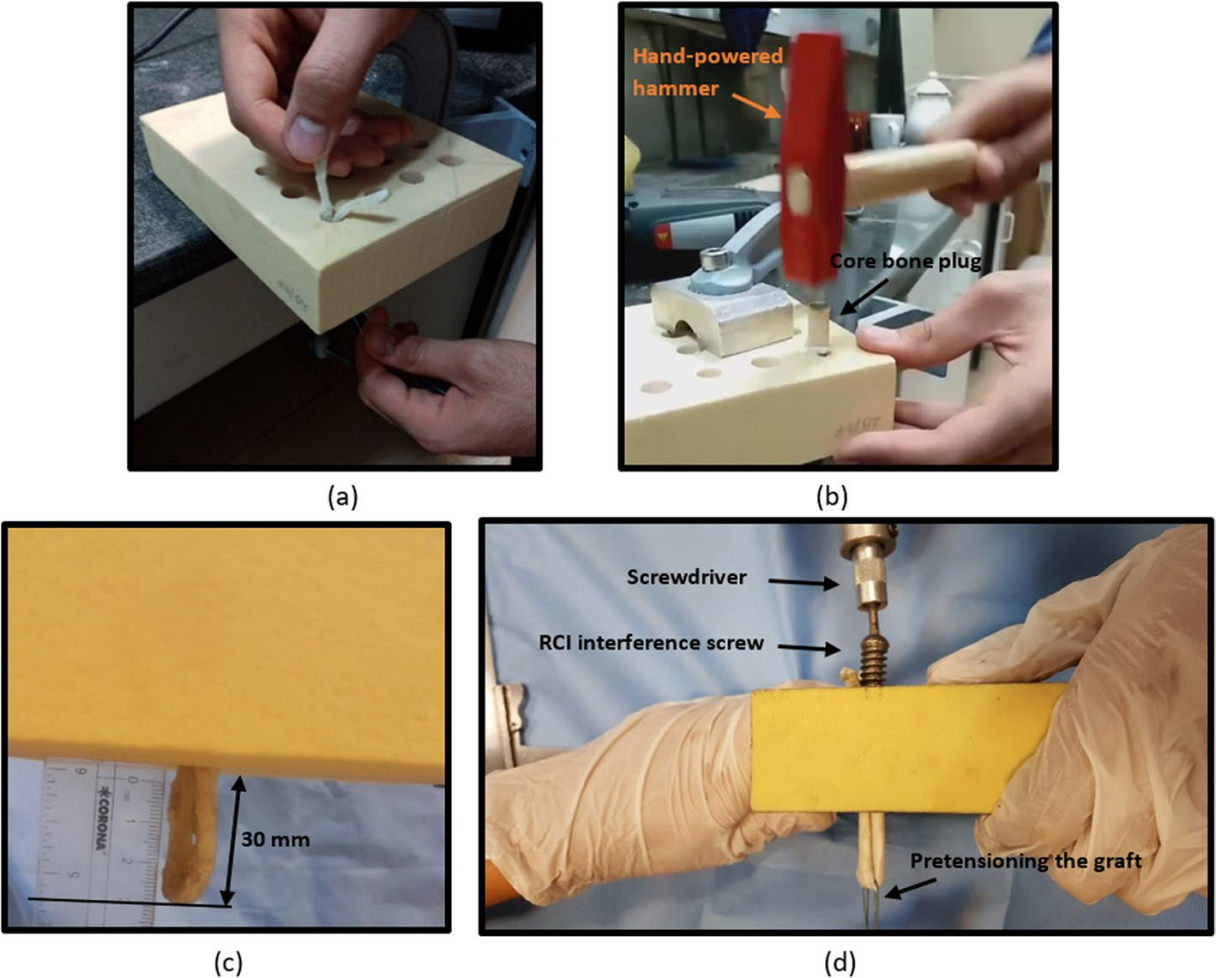
**a** Passing through a looped tendon into the tunnel with the aid of a guide suture. **b** The BASHTI insertion process of a core bone into a tunnel using a hand-powered hammer. **c** The gauge length of the looped tendon was maintained 30 mm, similar to the length of an intact ACL. **d** The insertion process of an interference screw into the tunnel using a screwdriver

After the drilling process, the tendon was prepared to be fixed into the tunnel. Initially, a looped tendon was passed through the tunnel with the aid of a guide suture (Fig. 3a). To make the core bone easier to insert into the tunnel, the edge of the core bone was chamfered. In the next step, the core bone was placed into the tunnel and forced in using a hand-powered hammer by applying strikes with a frequency of lower than 300 beats per minute (Fig. 3b) [25]. While the force was being applied to the core bone, the graft was pulled toward the opposite direction. This action produced a pretension into the graft, which is recommended for an ACL reconstruction [31]. After the insertion process was completed, the structure was ready for mechanical testing. It should be noted that in the insertion process, the gauge length of the looped tendon was kept 30 mm (Fig. 3c), similar to the length of an intact ACL [27].

### Interference screw insertion process

A reverse-thread interference (RCI) screw (7,209,413 10 × 30 mm RCI Screw) from Smith & Nephew Company (London, United Kingdom) with the outer diameter of 10 mm and total length of 32.5 mm (i.e., threaded length of 30 mm) was used to implement the screw insertion process. The geometry of the selected interference screw was in close agreement with a BASHTI core bone.

To prepare the samples, the looped tendon was passed through the tunnel, and the gauge length of the looped tendon was maintained 30 mm (i.e. it was left outside the tunnel) (Fig. 3c). Just like the BASHTI technique, the tunnel diameter was 10 mm. Similar to the previous practice, the screw was placed centrally between double-stranded graft and screwed in using a special screwdriver while the tendon graft was under a pretension (Fig. 3d). After the interference screw was fully inserted into the tunnel, the structure was ready for mechanical testing.

### Test setup

**Fig. 4:**
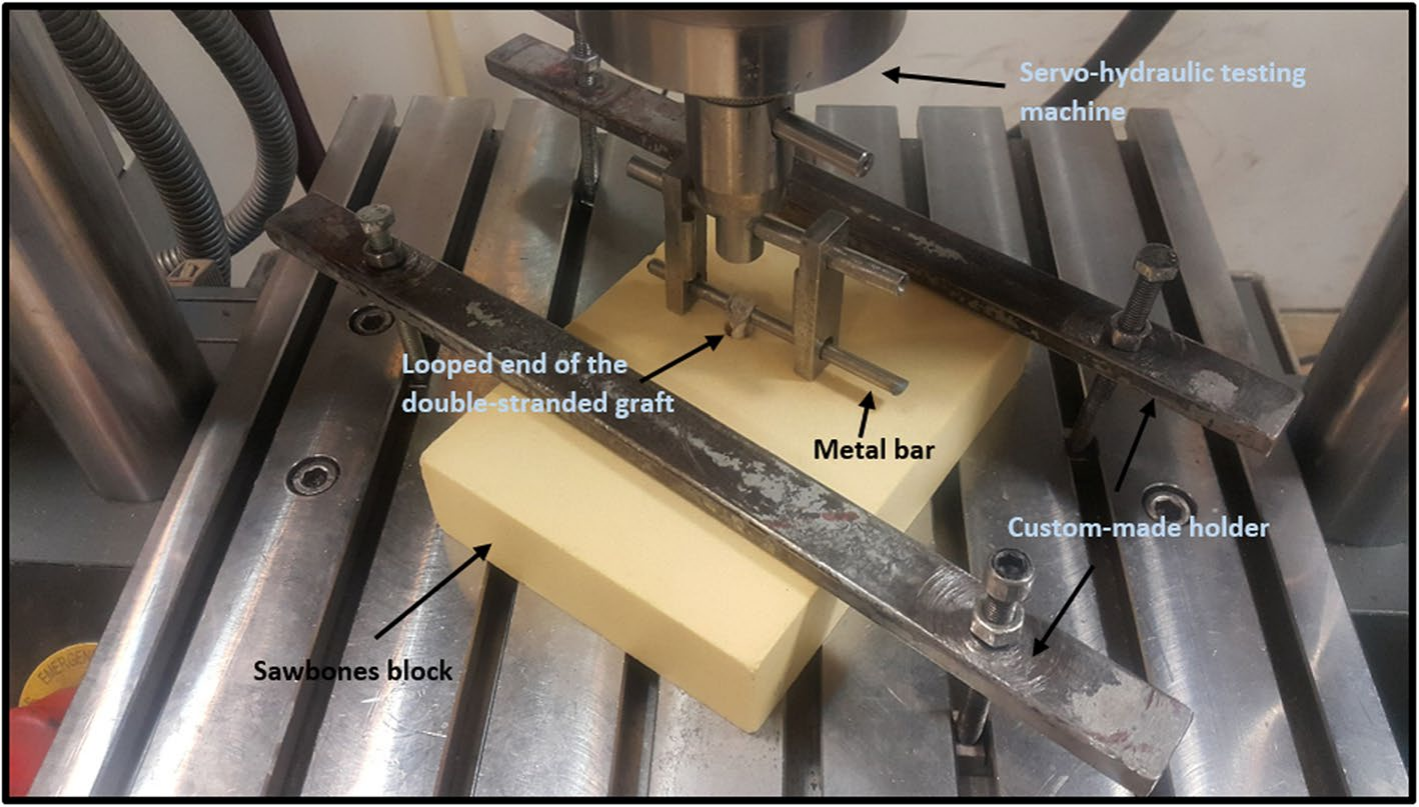
A typical test sample mounted on the mechanical testing machine

After fixing the graft into the tunnel, the experiments were performed on the structure using a servo-hydraulic testing machine (Amsler HCT 25–400; Zwick/Roell AG, Germany). In this regard, the Sawbones block was mounted onto the test machine using a premade mechanical setup. The block was fixed on the test machine so that the direction of the applied force became parallel to the tunnel axis. This condition represents the worst-case scenario for applying forces in a tibial tunnel. In the final step, the looped graft was hanged into the crosshead of the testing machine using a metal bar (Fig. 4).

The loading process consisted of three steps: a) preloading. In this step, a cyclic preconditioning load with a range of 10–50 N for 10 cycles with a frequency of 0.1 Hz was applied [32] to the sample. Preconditioning of viscoelastic materials like tendon graft is a standard procedure in tensile tests and validates the data in view of the history dependence characteristics of these materials [28]. In addition, the preconditioning makes collagen fibers straight in the direction of applied forces [33] and also eliminates the tendon loose length [32]. b) the main pull-out test. In the second step, a cyclic loading with a range of 50–200 N for 150 cycles with a frequency of 0.5 Hz was applied to the sample [34]. This step simulated the applied forces on the graft during flexion/extension at the early stage of the rehabilitation process after an ACL reconstructive surgery [34–37]. Also, the average forces applied to an intact ACL during normal walking and slope climbing are 169 N and 67 N, respectively [38]. c) the simple tensile test. After the cyclic loading, if the structure did not fail, the third step would be applied to measure the fixation strength of the structure. In this step, the specimens underwent a simple tensile loading with a loading rate of 20 mm/min until the failure of the structure [28, 29].

Two criteria were considered to conclude a failure has occurred; firstly, the displacement of the structure during loading stages should not exceed 10 mm, which is about 30% of the average intact ACL. After that, the graft might lose its functionality and result in an excessive laxity [39]. Secondly, if there are more than one peak points on the force-displacement graph, the second peak point would be selected as the failure load provided the drop after the first peak was within 10% of the first peak load. The small drop would represent a local micro fracture that could be healed/recovered in the body without affecting the whole structure [33]. The mode of failure was observed and recorded during the test process. Each test was repeated five times to ensure the repeatability of the results.

The average cyclic stiffness (ACS) was defined as follows to measure the functionality of the reconstructed graft under daily cyclic loads, especially in flexion/extension at the early stage of rehabilitation post ACL reconstruction:

1 $$ACS=\frac{F_C}{D_C\left/{N}_C\right.}\ \left(N\left/mm\right.\right)$$

where *F*_*C*_ is the amount of main cycling loading (i.e., the difference between the lower and upper values in the cyclical loading from 50 to 200 N which is 150 N), *D*_*C*_ is the pure displacement in the main cyclic loading (second loading step), and *N*_*C*_ is the number of completed cycles in the second loading step (i.e., 150 cycles).

In addition, the average pull-out stiffness (APS) was calculated by measuring the slope of the linear region of the force-displacement curve in the pull-out loading test.

### Statistical analysis methods

The 95% confidence intervals of the results were calculated using Student’s t distribution. Also, ANOVA one-way method (i.e., a statistical test that compares the variation in the group tests when one independent variable is used) was used to evaluate the recorded data. While, probability value (*P*-Value) was considered to determine whether the differences between results are significant or not. In case, if the *P*-Value is equal or less than 0.05, it means the differences between two groups with 95% confidence are significant.

## Results

**Fig. 5:**
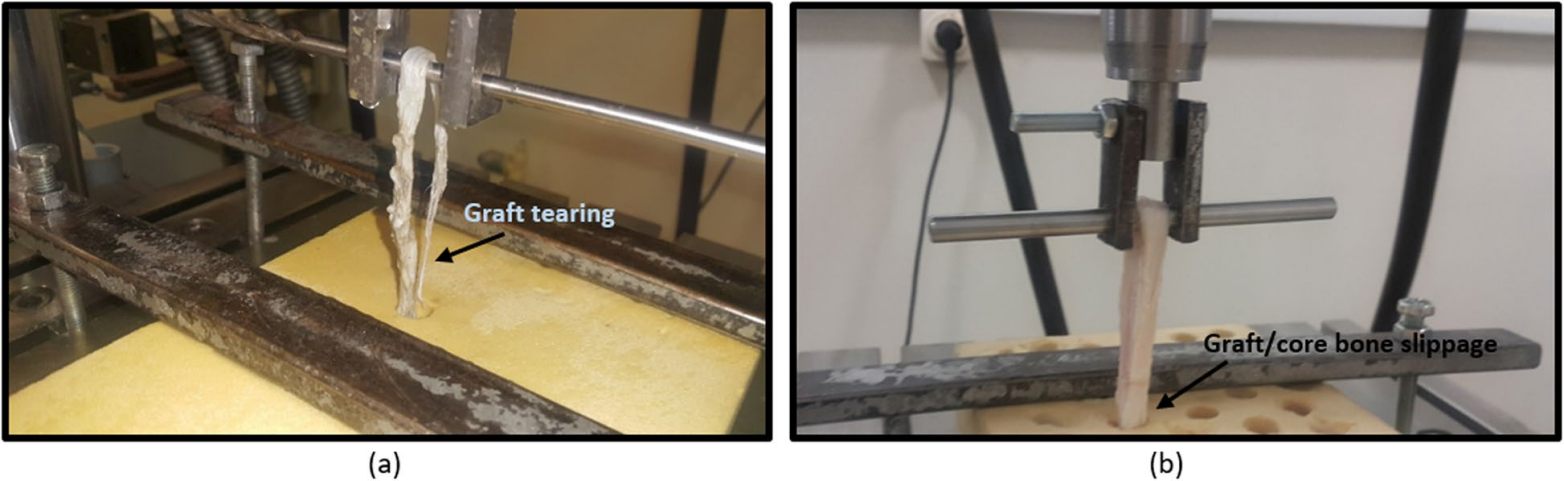
The failure modes observed in the experiments. **a** Rupture in tendon when using an interference screw. **b** Slippage in tendon when using BASHTI technique

All of the 10 specimens in the interference screw technique groups failed due to tearing of the graft. While, in the BASHTI technique, the failure occurred due to slippage of the graft/core bone at the fixation site in all of the 10 specimens. Figure 5 shows the two failure modes observed in this study.

Table 1 reports the modes of failure for the tested specimens.

**Table 1:** Mode of failure between different groups

Fixation Technique	Tendon Diameter (mm)	Slippage Failure (%)	Tearing Failure (%)
Interference screw	8	0	100
Interference screw	9	0	100
BASHTI	8	100	0
BASHTI	9	100	0

For those specimens which successfully passed the two steps of cyclical and pull-out loading, the failure loads were recorded.

**Fig. 6:**
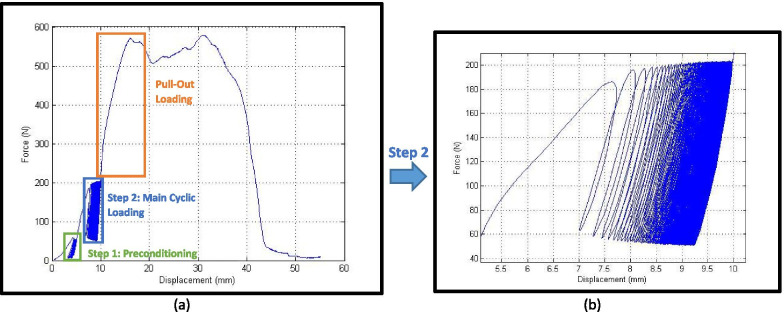
**a** A typical load-displacement graph when a 9 mm tendon graft was used. **b** Hysteresis loops become stable at the end of main cyclical loading

In Fig. 6 a typical load-displacement graph for interference screw technique is seen, which consists of three aforementioned steps in the loading condition. Also, the magnified step 2, main cyclic loading, is shown in Fig. 6b. As shown in Fig. 6a, there are two peak points on the graph, but considering the criteria mentioned in section 2.6, the first peak was taken as the failure load.

The mechanical properties of all groups are shown in Table 2.

**Table 2:** Mechanical properties of all tested groups including fixation strength, average cyclic stiffness (ACS), and average pull-out stiffness (APS)

Fixation Technique	Tendon Diameter (mm)	Fixation Strength (N)	ACS (kN/mm)	APS (N/mm)
Interference Screw	8	439 ± 132	10.3 ± 5.3	109 ± 40
Interference Screw	9	549 ± 87	10.3 ± 4.7	91 ± 13
BASHTI	8	360 ± 123	3.3 ± 0.6	79 ± 27
BASHTI	9	278 ± 103	2.4 ± 1.2	111 ± 40

## Discussion

### Stable condition of hysteresis loops

In Fig. 6b, a typical output of a cyclic loading test was shown. As demonstrated, the tendon behaved like a viscoelastic material with hysteresis loops, indicating a time dependency characteristic. As shown in the graph, the hysteresis loops gradually became thinner and thinner which implies the energy loss due to the internal friction between the tendon fibers reached its minimum. This means the graph reached its stable condition, and no more creep was observed on the specimen [33]. After the cyclical step became stable, a pull-out loading was applied to the sample.

### Mode of failure

**Fig. 7:**
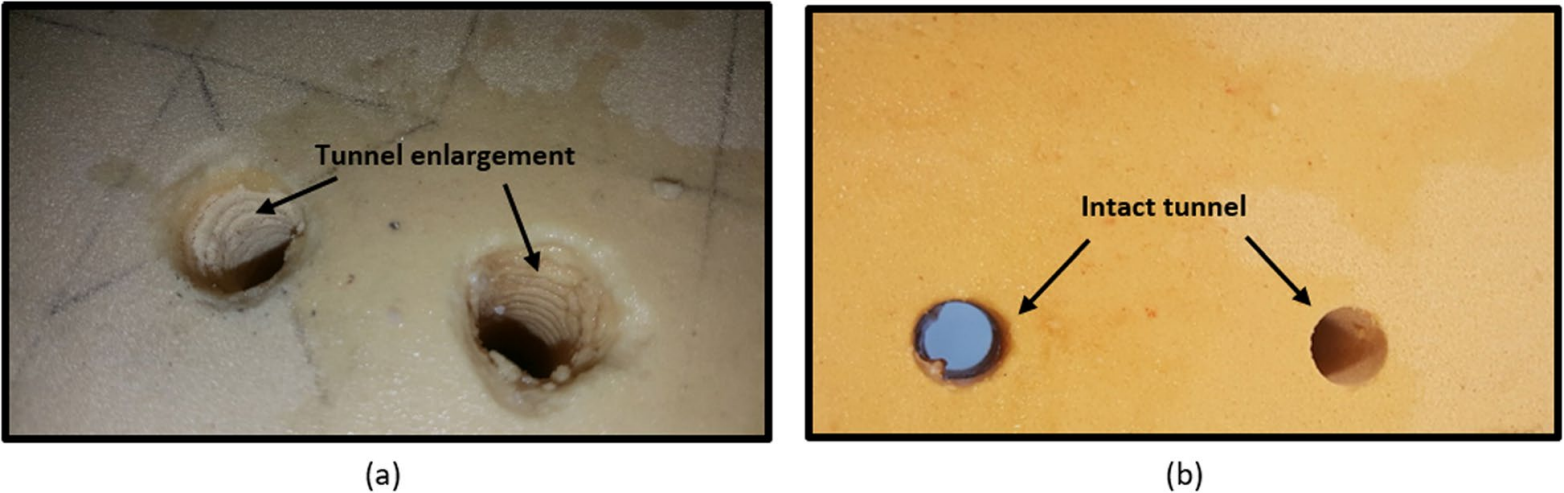
The scare of interference screw and core bone on the tunnel wall; **a** Massive tunnel enlargement was seen after the fixation process using the interference screw technique. **b** The tunnels remained intact after fixation using BASHTI technique

As shown in Table 1, all the samples with interference screws failed due to tendon rupture, while all the specimens tested on BASHTI technique failed as a result of slippage. The differences in failure modes of the two techniques can be referred to their fixation structures. With the interference screw method, the sharp edges of the screw may squeeze/cut the bone and tendon graft during the insertion into the tunnel, influencing the strength of the fixation in the pull-out examination. On the other hand, in the BASHTI technique, the bone plug does not have any sharp edges, and therefore, the tendon fibers did not encounter the damage that occurred in the interference screw technique. However, unlike an interference screw method in which the screw threads maintain the friction at the contact zone, the BASHTI core bone would provide a lower friction at the fixation region. In other words, the reason for the failure in BASHTI technique is due to weakness in the fixation structure, rendering a slippage of the core bone/tendon at the fixation site. Figure 7a shows how the interference screw threads cut/damage the tunnel wall. This may also lead to a tunnel widening which is another problem when an interference screw is used [9, 10]. While Fig. 7b shows that a BASHTI tunnel is intact even after the reconstruction and, therefore, no tunnel widening was observed.

### Effect of tendon diameter on fixation properties

**Fig. 8:**
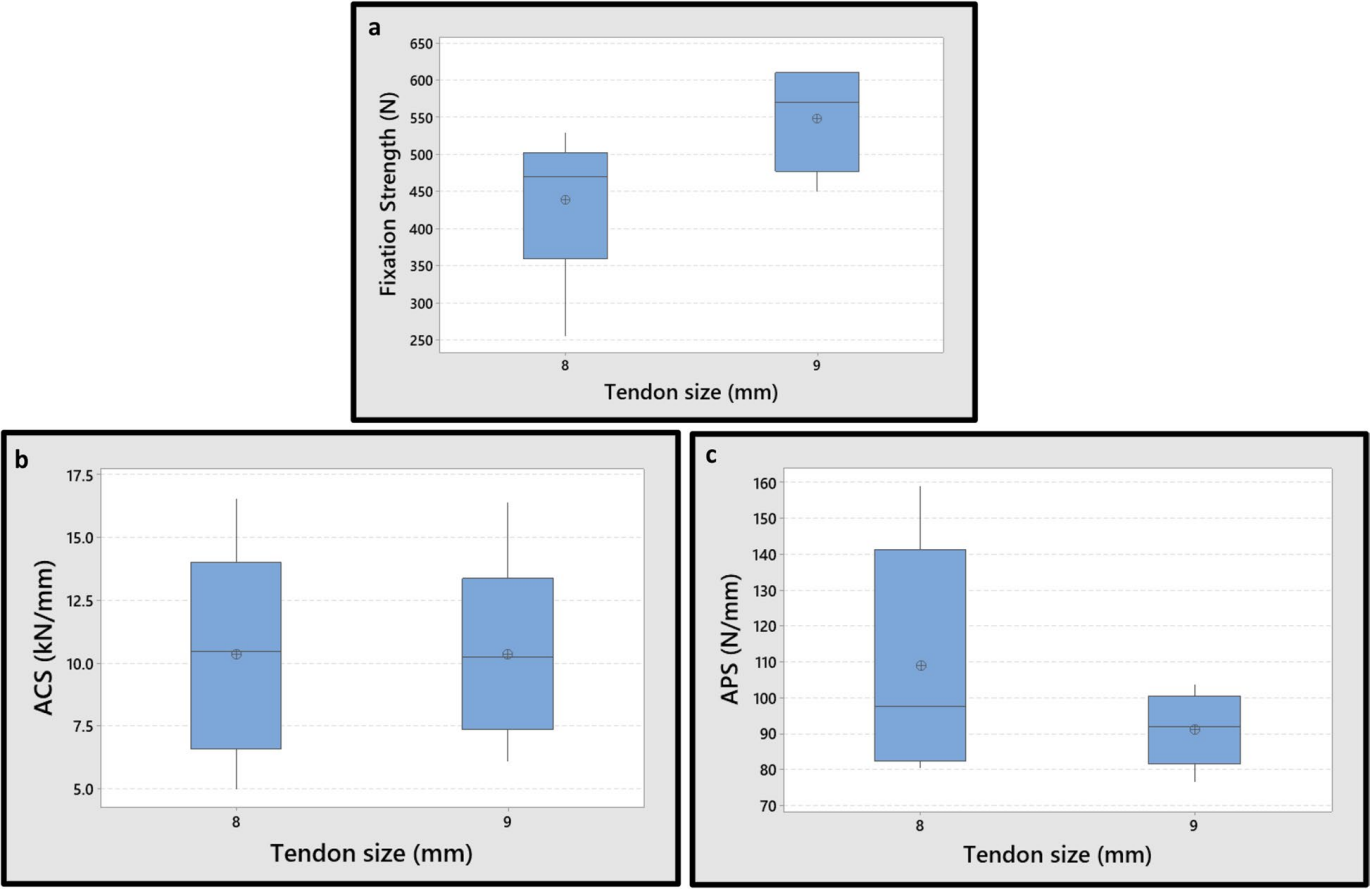
Effect of tendon diameter on mechanical properties for the interference screw technique; **a** The fixation strength. **b** Average cyclic stiffness (ACS). **c** Average pull-out stiffness (APS). Differences of the mechanical properties between two graft sizes in the interference screw technique were N.S. (*P*-Value> 0.05)

In the interference screw groups, the average fixation strength of the 9 mm tendon was 25% higher than that of the 8 mm tendon, due to higher friction in the contact zone. In other words, the screw threads created higher pressure in 9 mm tendon groups. However, the differences were not significant (N.S.), with 95% of confidence interval (*P*-Value = 0.089). Also, the average ACS values of these two groups were almost the same (*P*-Value = 0.999). The APS is related to elastic deformation of the structure and represents the resistance of the reconstructed graft to sudden impact loadings. In addition, the differences between the APS values of 8 mm and 9 mm tendons were N.S., with 95% of confidence interval (*P*-Value = 0.277). As a result, no significant influence of tendon diameter on the mechanical properties of the fixation was observed for the interface screw technique (Fig. 8).

**Fig. 9:**
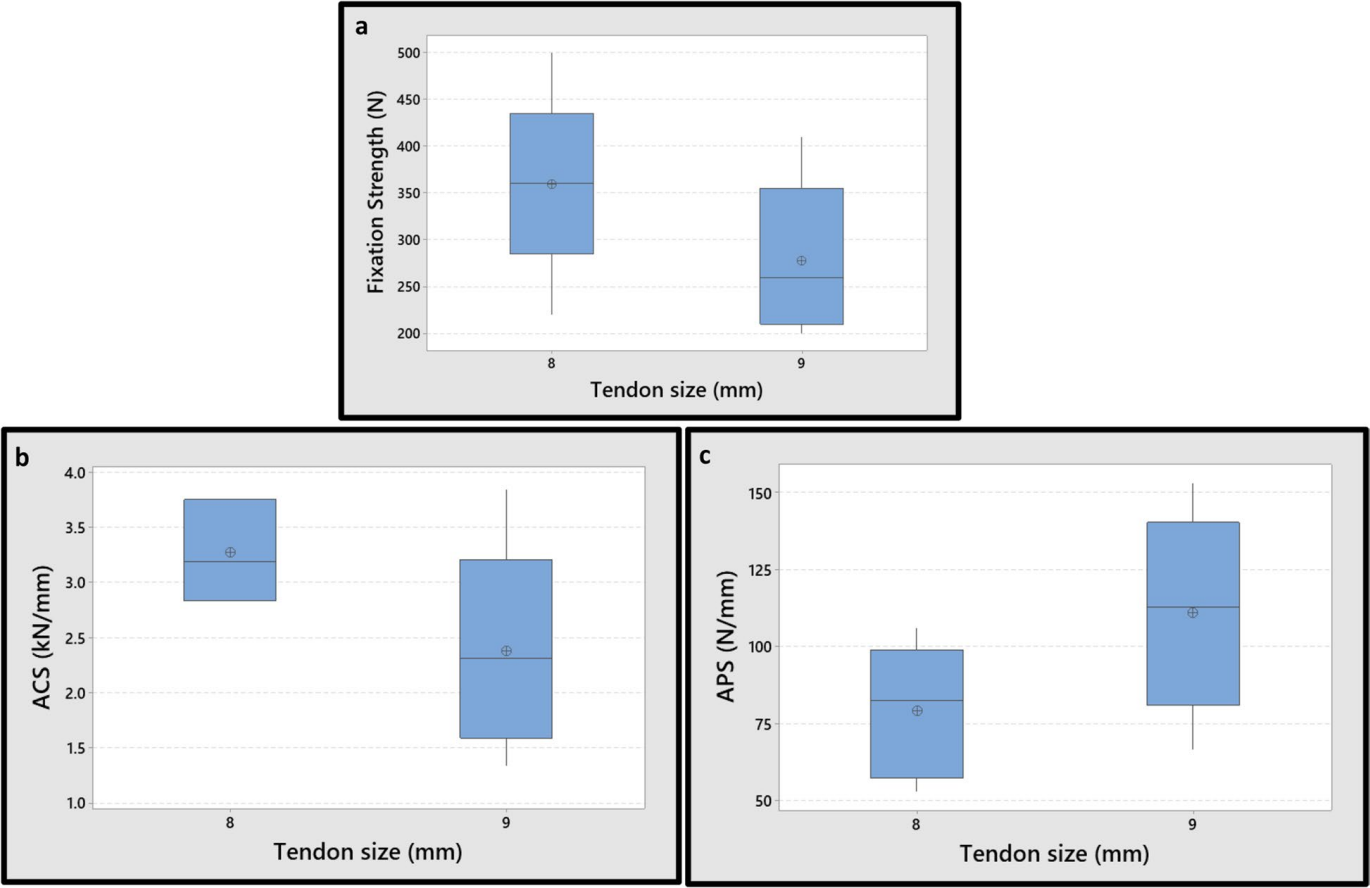
Effect of tendon diameter size on mechanical properties for the BASHTI technique; **a** The fixation strength. **b** Average cyclic stiffness (ACS). **c** Average pull-out stiffness (APS). Differences on the mechanical properties between two graft sizes in the BASHTI technique were N.S. (*P*-Value> 0.05)

In the BASHTI groups, the average fixation strength of the 9 mm tendon was 23% lower than that of the 8 mm tendon. The over-compression of the tendon grafts, and excessive damages to the graft in the larger diameter, is believed to be responsible for the decrease in the fixation strength from 8 to 9 mm tendons in the BASHTI technique. However, the differences were N.S., with 95% of confidence interval (*P*-Value = 0.195). Also, the differences between the ACS and APS values of 8 mm and 9 mm tendons were N.S., with 95% confidence interval (the *P*-Values were 0.095 and 0.104 for the ACS and APS, respectively) (Fig. 9).

Therefore, it was concluded that the variation in the diameter of the tendon had no significant effect on the mechanical properties of both techniques.

### Effect of reconstruction technique on fixation properties

**Fig. 10:**
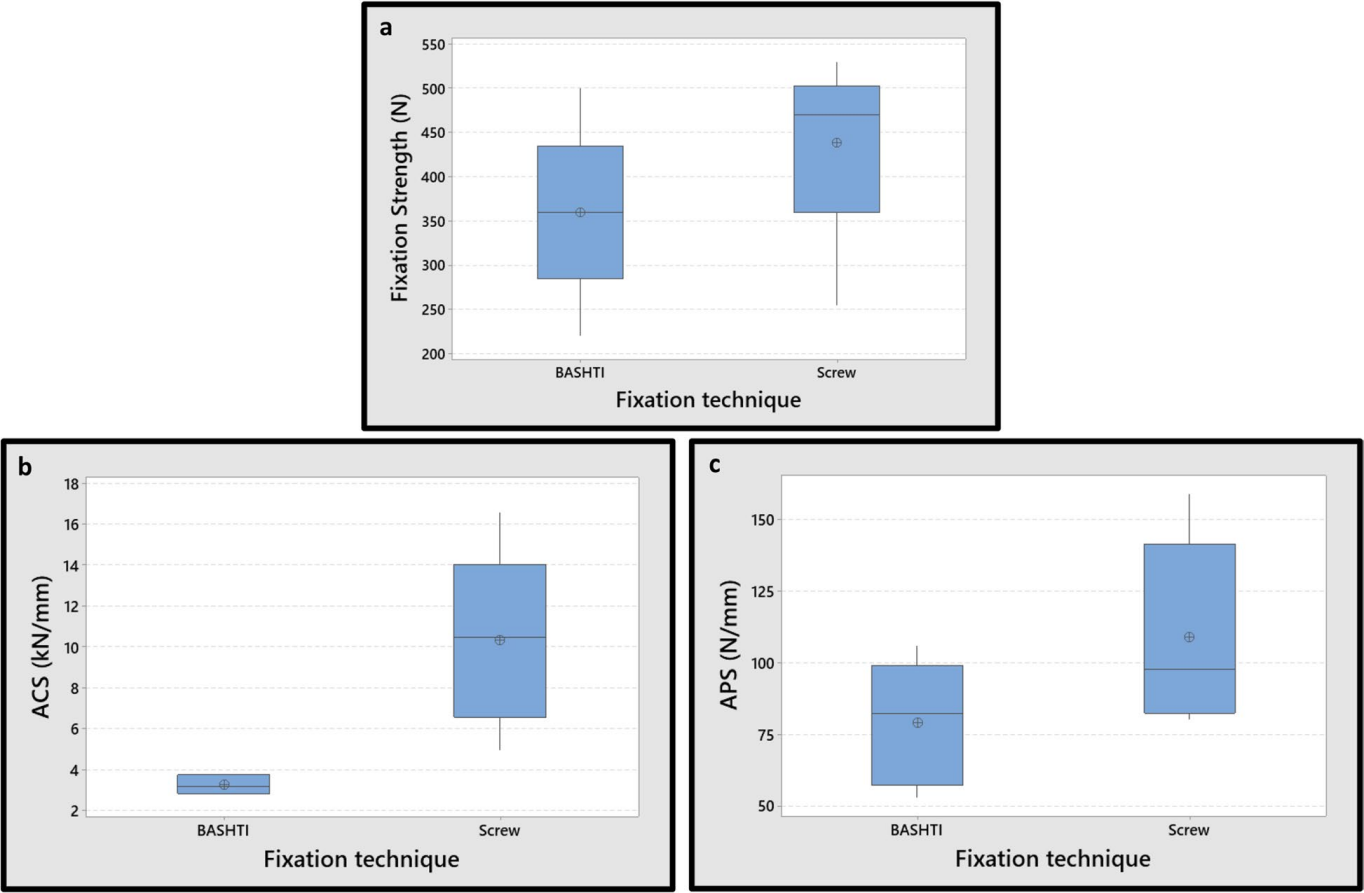
Effect of fixation technique on mechanical properties for 8 mm tendon graft size; **a** The fixation strength. **b** Average cyclic stiffness (ACS). **c** Average pull-out stiffness (APS). Differences between fixation strength and APS of two techniques in 8 mm tendon graft size were N.S. (*P*-Value> 0.05)

The results obtained indicated that there was N.S. difference between the fixation strength and APS of two techniques for the 8 mm tendon diameter groups (*P*-Value = 0.259 and 0.125 for fixation strength and APS, respectively). Although, the discrepancy in the ACS values of the two techniques was considerable (*P*-Value< 0.01) for the 8 mm tendon diameter (Fig. 10). In other words, the ACS in the interference screw technique was notably higher than that in the BASHTI technique. It implies that the interference screw method had a higher resistance against cyclic forces. This is mainly because the screw threads gripped the tendon more strongly than did the core bone in BASHTI technique. The ACS means the resistance of the tendon graft to the displacement in cyclic loading. When the tendon fibers were completely trapped within the screw threads, there was no chance of excessive displacement in the cyclic loading at the fixation site. Consecutively, when the displacement became extremely lower, the stiffness became higher. On the other hand, since there was no thread in the core bone in BASHTI groups, the structure encountered a weak resistance to displacement at cyclic loading. Therefore, lower values of ACS were observed in BASHTI groups with respect to the interface screw technique.

**Fig. 11:**
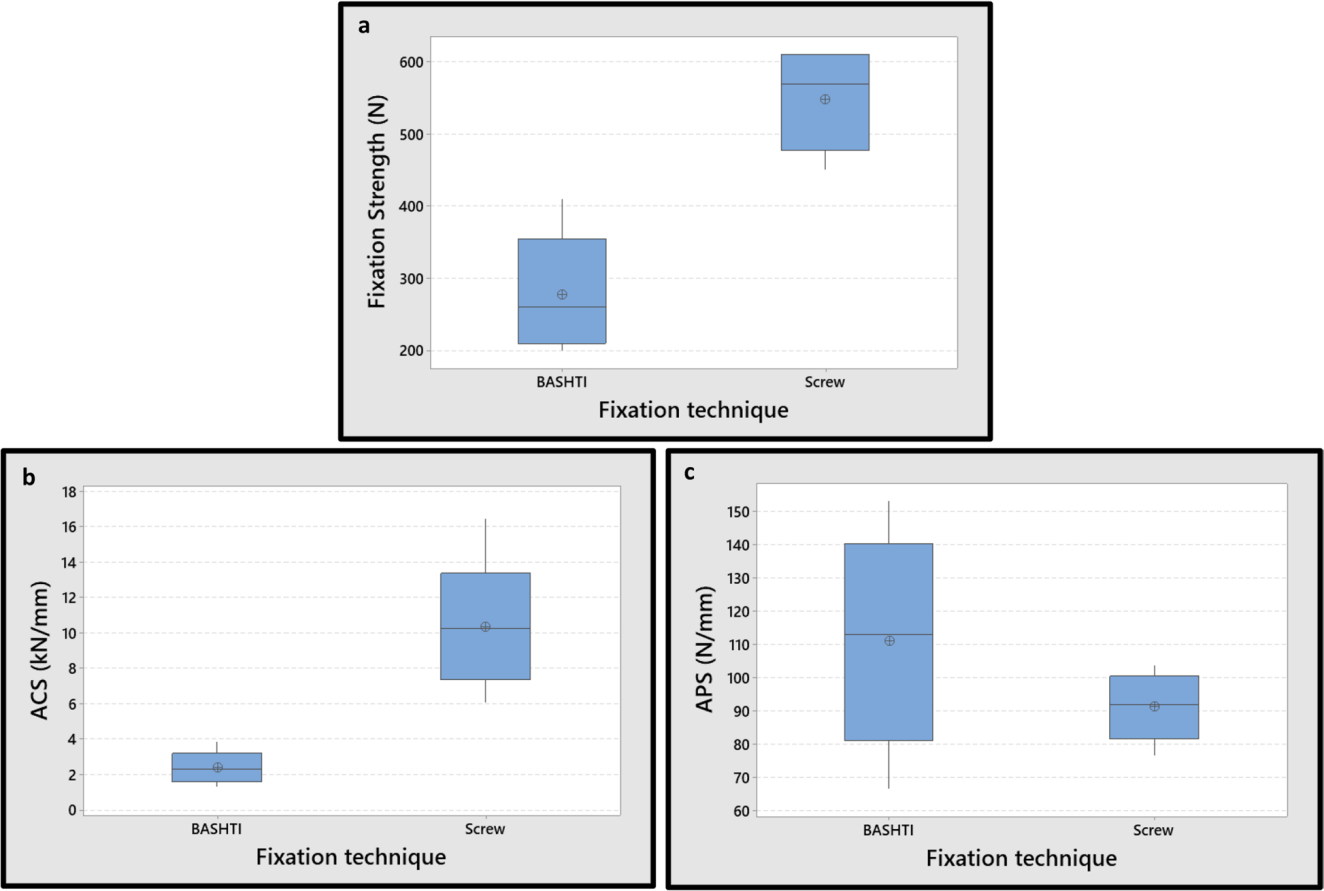
Effect of fixation technique on mechanical properties for 9 mm tendon graft size; **a** The fixation strength. **b** Average cyclic stiffness (ACS). **c** Average pull-out stiffness (APS). While the fixation strength and ACS differences between two techniques in 9 mm tendon graft size were significant (*P*-Value< 0.01), the differences in the APS between two techniques were N.S. (*P*-Value> 0.05)

In the 9 mm tendon diameter groups, the differences between fixation strength and ACS values of two techniques were remarkable (*P*-Value = 0.001, 0.002 for fixation strength and ACS, respectively). Although the average APS values in BASHTI technique were higher than interference screw technique, the differences between APS values of two groups were inconsiderable (*P*-Value = 0.229) (Fig. 11).

As mentioned above, the difference in the fixation strength in 8 mm tendon between two techniques were insignificant, while it was significant for the 9 mm tendon. This size-dependent difference of the strength for two techniques is hypothesized to be due to the over-compression which occurred in the 9 mm tendon. In the BASHTI technique, the compression in the tunnel increased excessively for the larger tendon size since the tunnel diameter was fixed (i.e. 10 mm). As a result, the extensive compression squeezed and damaged the tendon in the transverse direction and made its mechanical properties weaker. Also, there was no thread in the core bone to increase the friction force. Therefore, either the tendon or core bone slipped out of the tunnel. On the other hand, despite of existence of the over-compression, the friction was higher in the 9 mm tendon groups of the interface screw method (due to the existence of threads). As a result, the fixation strength in 9 mm tendon became higher than that of the 8 mm tendon in the interference screw groups. To summarize, by increasing the tendon diameter in the interference screw technique, the fixation strength increased, while in the BASHTI technique, the fixation strength decreased.

### Overall comparison of interface screw and BASHTI techniques

The ACS value, which is determined in the main cyclic loading, represents the resistance of the tendon itself against the forces applied on it during the knee’s flexion/extension in the early stage of the rehabilitation [34, 35, 37]. Since the ACS was higher in the interface screw technique with respect to the BASTHTI method for both 8 and 9 mm tendon diameters, the patients operated using the BASHTI technique might need to reduce active rehabilitation at the early stage after surgery, have more precaution in the early stage of recovery, and be careful of full load-bearing activities (e.g., heavy sports such as soccer) in this period.

As discussed previously, the interference screw technique had a higher fixation strength as the screw threads anchored the tendon at the tunnel. While, in the BASHTI technique, the friction force was lower than that in the interference screw groups, and it resulted in a smaller fixation strength. Therefore, it is proposed to modify the design of the BASHTI core bone to make it spikier (e.g., by using a sheath).

Noteworthy, the fixation strengths of both groups were comparable, and both were greater than the average forces applied to an intact ACL during normal walking (i.e., 169 N), or slope climbing (i.e., 67 N) [38]. As a result, considering a restricted early rehabilitation, the BASHTI technique could be more beneficial because of its cost-effectiveness, fewer side effects, and inflammatory responses, and no more interfering in imaging post-operation. Also, since this technique is organic and uses the same patient bone and soft tissue, the healing process would be faster. Finally, implementing the BASHTI technique on a live animal model would be necessary to examine this method in actual conditions.

## Conclusions

In this study, the mechanical properties of ACL reconstruction in two different techniques, i.e., the interference screw and BASHTI techniques, with two different tendon graft diameters were investigated. The results showed that although mechanical properties of the BASHTI technique were lower compared to the inference screw method, this technique could be used as an alternative technique in an ACL reconstruction due to its clinical advantages and lower costs. The study concluded that 8 mm tendon group is recommended for the BASHTI technique. Using human grafts and bones in experimentation and in-vivo animal model testing can be suggested for future work.

## Data Availability

The datasets used and/or analysed during the current study are available from the corresponding author on reasonable request.
